# Comparison of Surgical Outcomes Between Separation Surgery and Piecemeal Spondylectomy for Spinal Metastasis: A Retrospective Analysis

**DOI:** 10.3389/fsurg.2021.686930

**Published:** 2021-11-22

**Authors:** Lun Xu, Wending Huang, Weiluo Cai, ZhengWang Sun, Meng Fang, Yingzheng Ji, Shuoer Wang, Jianing Zhang, Tu Hu, Mo Cheng, Wangjun Yan

**Affiliations:** ^1^Department of Musculoskeletal Oncology, Fudan University Shanghai Cancer Center, Shanghai, China; ^2^Department of Oncology, Shanghai Medical College, Fudan University, Shanghai, China; ^3^Department of Orthopedic, Naval Medical Center of PLA, Second Military Medical University, Shanghai, China

**Keywords:** spinal metastasis, separation surgery, piecemeal spondylectomy, surgical outcomes, retrospective analysis

## Abstract

**Objective:** This study aimed to compare the outcomes between piecemeal spondylectomy and separation surgery for patients with spinal metastasis.

**Summary of Background Data:** Piecemeal spondylectomy and separation surgery are two widely-used treatment options for spinal metastasis. However, no studies have compared the surgical outcomes between both treatment modalities.

**Methods:** Patients with spinal metastasis who underwent piecemeal spondylectomy or separation surgery between August 2017 and April 2020 at our spine center were recruited. Demographic, preoperative, perioperative, and follow-up data were collected and analyzed. Kaplan–Meier analysis and the log-rank test were used to analyze overall survival (OS) and progression-free survival (PFS) in patients with spinal metastasis.

**Results:** Overall, 26 patients were treated with piecemeal spondylectomy, and 29 underwent separation surgery with postoperative stereotactic radiosurgery. Both groups showed significant postoperative improvements in neurological status. The piecemeal spondylectomy group had significantly more blood loss (1784.62 ± 833.64 vs. 1165.52 ± 307.38 ml) and required longer operative time (4.76 ± 0.93 vs. 3.73 ± 1.15 h) than the separation surgery group. No significant difference in OS was found between the groups (*P* = 0.064); however, patients in the separation surgery group experienced less local recurrence than those in the piecemeal spondylectomy group (*P* = 0.0014). Notably, significant differences were detected in the development of complications between the groups (*P* = 0.029).

**Conclusion:** Separation surgery led to less blood loss and reduced complications and had shorter operation time than piecemeal spondylectomy. Although no significant differences were found in OS between the groups, separation surgery was associated with better PFS compared with piecemeal spondylectomy. These findings suggest that separation surgery has some advantages over piecemeal spondylectomy for patients with spinal metastatic disease.

## Introduction

The spine is the most preferred site of metastatic skeletal involvement (1), and spinal metastasis usually leads to impairment of spinal structure and spinal cord compression (10%), causing substantial pain, hypercalcemia, pathological fractures, spinal instability, and even paralysis (1, 2). Surgical intervention is often required and proved to be effective in patients with unstable metastatic lesions or neurologic impairment (3, 4). However, surgical management of spinal metastasis is particularly difficult due to complex anatomical structures and involvement of important adjacent nerves and vessels.

Total spondylectomy refers to the complete surgical removal of metastatic lesions to achieve curative surgical resection. According to the classification of the Global Spine Tumor Study Group (GSTSG) (5), total vertebrectomy can be achieved by either an en bloc or piecemeal manner. Tomita et al. (6) first proposed total en bloc spondylectomy for the treatment of patients with solitary spinal metastasis. This technique involves en bloc laminectomy with posterior instrumentation and total corpectomy, followed by anterior column reconstruction. However, the application of the en bloc technique is usually limited due to the complex anatomical structure of the spine (7). In addition, for patients requiring radical surgical resection, piecemeal spondylectomy is generally performed for radical purposes. However, radical resection of lesions is often accompanied by surgical trauma and a high rate of complications (8). Therefore, a careful preoperative assessment is needed to determine whether the patients are suitable for total spondylectomy.

Stereotactic radiosurgery (SRS) is a non-invasive and targeted treatment modality that delivers a high radiation dose to the metastatic lesion without damage to surrounding tissues, thereby achieving good local control (9, 10). SRS has been applied in radio-insensitive tumors, such as melanoma and renal cancer, due to the high radiation dose (11). Molding et al. (12) first proposed the concept of “separation surgery,” in which a limited tumor resection and segmental instrumentation are performed to provide decompression of the spinal cord and a tumor-free margin. Indications of separation surgery include Metastatic Epidural Spinal Cord Compression, pathological fracture, and spinal instability (13). The goal of separation surgery is to improve the application and efficacy of SRS in metastatic spinal tumors.

Although spondylectomy and separation surgery have been widely used for treatment of spinal metastatic tumors, few studies have compared outcomes between these two different surgical modalities. In this study, we retrospectively reviewed the data of patients who underwent piecemeal spondylectomy or separation surgery in our institution to compare surgical outcomes between these two treatment modalities.

## Materials and Methods

### Patients

Data of patients with spinal metastasis who underwent piecemeal spondylectomy or separation surgery between April 2017 and April 2020 at our institution were retrospectively reviewed, and 55 patients were included in our study cohort. According to the treatment modality, patients were divided into the following two groups: piecemeal spondylectomy (26 patients) and separation surgery (29 patients). Inclusion criteria were as follows: (1) histological diagnosis confirmed by biopsy or postoperative pathology; (2) surgery at our spinal tumor center; and (3) complete information of imaging data, laboratory examination data, and medical records. Exclusion criteria were as follows: (1) incomplete resection or loss to follow-up; (2) hematological tumors, except multiple myeloma, in the spine; or (3) those who did not receive SRS after surgery in the separation surgery group. The selection criteria for piecemeal spondylectomy include: (1) solitary spinal metastasis; (2) Severe destruction of anterior column cannot be stabilized by posterior decompression alone and posterior fixation. As for separation surgery + SRS, the selection criteria include: (1) Patients with epidural spinal cord compression (Grade >2); (2) Radioresistant or previously radiated tumor. This study was approved by Ethics Committee of the Shanghai Cancer Center. In accordance of the Declaration of Helsinki, informed consent was obtained from all patients.

### Data Collection

Baseline characteristics of patients, including age, sex, type of primary tumor, and main level of metastasis, were recorded. Various criteria were used to assess the patients' status. The Frankel score (14) was used for the quantification of neurological status. The patients' physical ability was assessed using the revised Tokuhashi score (15) and Karnofsky score (16). The choice of surgical strategy was based on the Tomita score (17). Perioperative factors included operation time, intraoperative blood loss, type of procedure, and methods of reconstruction, and postoperative factors included neurological status, complications, and radiotherapy after surgery. Outcome factors, including overall survival (OS), local recurrence, and changes in neurological status, were compared between the two groups. Due to the high radiation dose of SRS, patients with radiation-resistant cancer were also included in the separation surgery group.

In our study, Follow-up was performed by telephone calls and outpatient review until death. Follow up was conducted at an initial 3 months postoperative outpatient visit in the first year, followed by at least 6 months outpatient imaging in the second to third year. The metastatic lesions were evaluated by at least one type of imaging examination, such as CT, MRI, PET/CT. OS was defined as the interval between the date of surgery to patient death, while progression-free survival (PFS) was defined as the time from the date of surgery to the occurrence of local recurrence or patient death.

### Surgical Techniques

In piecemeal spondylectomy, the patients were placed in a prone position for pedicle screw placement. After pedicle screws were inserted into the upper and lower vertebral pedicles, the vertebral appendix and pedicle were removed with nucleus pulposus forceps and rongeur. The pathological vertebrae were piecemeal resected after posterior instrumentation. The anterior spinal column was reconstructed with a titanium mesh filled with autologous bone or (and) allogeneic bone.

In separation surgery, the lamina was first removed with a grinding drill and a rongeur. The resection extent included the epidural tumor and part of the normal adjacent areas. After removal of the bilateral articular processes and pedicle, the epidural part of the tumor and the posterior longitudinal ligament were resected to achieve complete decompression around the canal and a 2–3 mm gap between the tumor and the spinal cord. If the posterior part of the vertebral body required resection, a suitable titanium mesh should be placed between the upper and lower vertebral bodies to complete anterior column reconstruction, especially when more than 50% of the vertebral body was involved. Then, the pedicle screws were placed at two segments above and below the affected vertebra, respectively, and internal fixation was performed. Finally, important structures, such as the spinal cord and nerve roots, were separated from the tumor tissue by surgery. SRS is generally performed in 30 days after separation surgery. The SRS radiotherapy regimen was high-dose hypofractionated SRS (24–30 Gy/3–4 times). The specific regimen of the radiotherapy was based on preoperative tumor volume, postoperative imaging data, history of previous radiotherapy, and paravertebral involvement.

### Statistical Analysis

The paired *t*-test and Mann-Whitney test were used to compare the continuous variables (Age, Blood loss, Operative time) at baseline between the piecemeal spondylectomy and separation surgery groups, while the chi-square test (Sex, Frankel grade, Karnofsky score, Tomita score, Revised Tokuhashi score, Primary site, Complications) was used for the comparison of categorical data. The Kaplan–Meier method was used to evaluate postoperative survival, and differences in survival curves were analyzed by the log-rank test. *P* < 0.05 (two sides) was considered statistically significant. All statistical analyses were performed using SPSS (version 21.0, IBM) and R software (version 3.6.1).

## Results

### Baseline Characteristic of Patients

From April 2017 to April 2020, 55 patients with spinal metastasis were treated with piecemeal spondylectomy or separation surgery + SRS at our institution. All patients in the separation surgery group completed the scheduled SRS protocol (high-dose hypofractionated SRS; 24–30 Gy/3–4 times). Detailed information on patient characteristics is listed in Table 1. No significant differences were detected in preoperative factors between the two groups.

**Table 1:** Baseline characteristics of patients.

	**Separation surgery + SRS**	**Piecemeal spondylectomy**	***P*-values**
Number of patients	29	26	
Sex			0.237
Male	18 (62.06%)	12 (46.15%)	
Female	11 (37.93%)	14 (53.85%)	
Age (years)	56.31 ± 10.28	58.27 ± 10.85	0.231
Preoperative Frankel grade			0.371
A	1 (3.45%)	1 (3.85%)	
B	2 (6.90%)	2 (7.69%)	
C	4 (13.79%)	7 (26.92%)	
D	14 (48.28%)	14 (53.85%)	
E	8 (27.59%)	2 (7.69%)	
Preoperative Karnofsky score			0.117
80–100	20 (68.97%)	17 (65.38%)	
50–70	8 (27.59%)	9 (34.62%)	
0–50	1 (3.44%)	0 (0%)	
Tomita score			0.211
2–3	7 (24.14%)	9 (34.62%)	
4–5	12 (41.38%)	9 (34.62%)	
6–7	5 (17.24%)	7 (26.92%)	
8–10	5 (17.24%)	1 (3.84%)	
Revised Tokuhashi score			0.343
0-8	11 (37.93%%)	5 (19.23%)	
9–11	12 (41.38%)	13 (50%)	
12–15	6 (20.69%)	8 (30.77%)	
Primary site			0.44
Breast	5 (17.24%)	5 (19.23%)	
Lung	8 (27.59%)	3 (11.54%)	
Prostate	3 (10.34%)	1 (3.85%)	
Liver	3 (10.34%)	1 (3.85%)	
Thyroid	1 (3.45%)	3 (11.54%)	
Renal	2 (6.90%)	3 (11.54%)	
Others	7 (24.13%)	10 (38.46%)	
Location of spinal metastasis			0.066
Cervical	2 (6.90%)	4 (15.38%)	
Thoracic	15 (51.72%)	14 (53.85%)	
Lumbar	9 (31.03%)	8 (30.77%)	
Sacral	3 (10.34%)	0 (0.00%)	

### Perioperative Data and Outcomes

**Figure 1:**
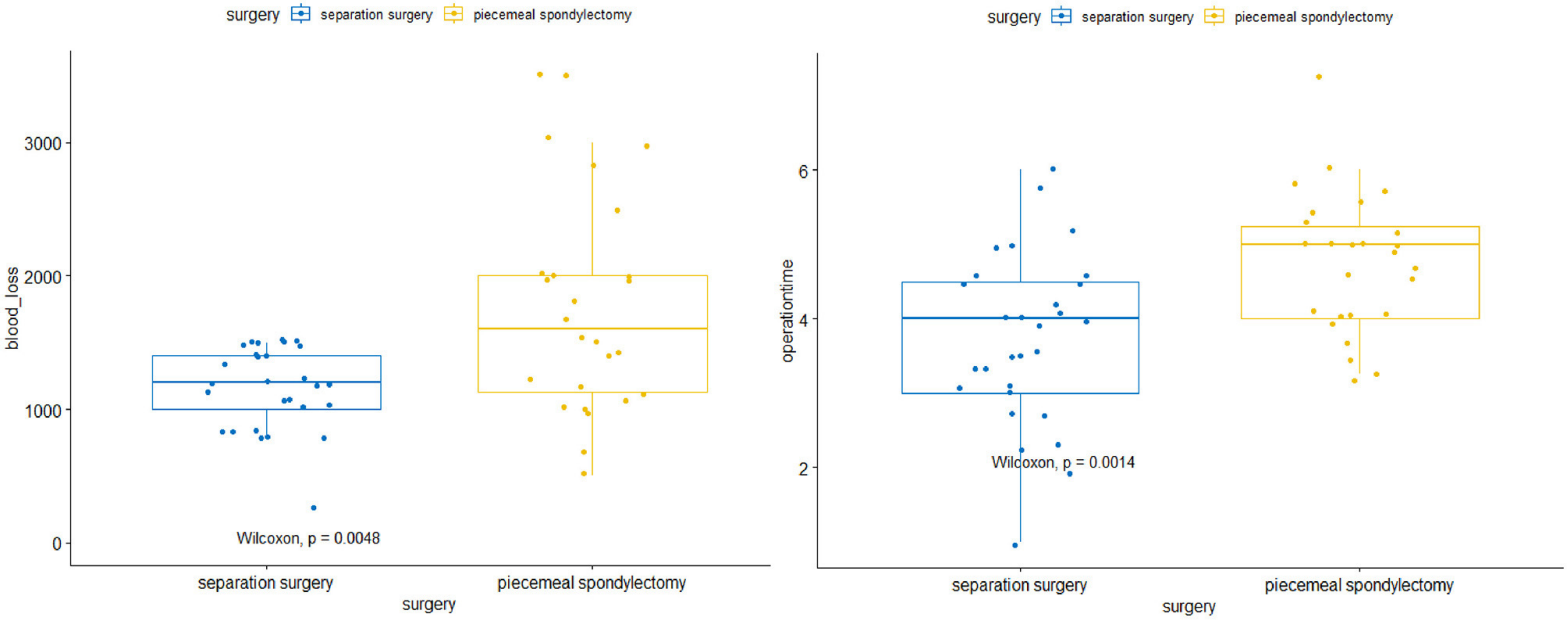
Comparisons of intraoperative blood loss and operative time between separation surgery and piecemeal spondylectomy.

**Figure 2:**
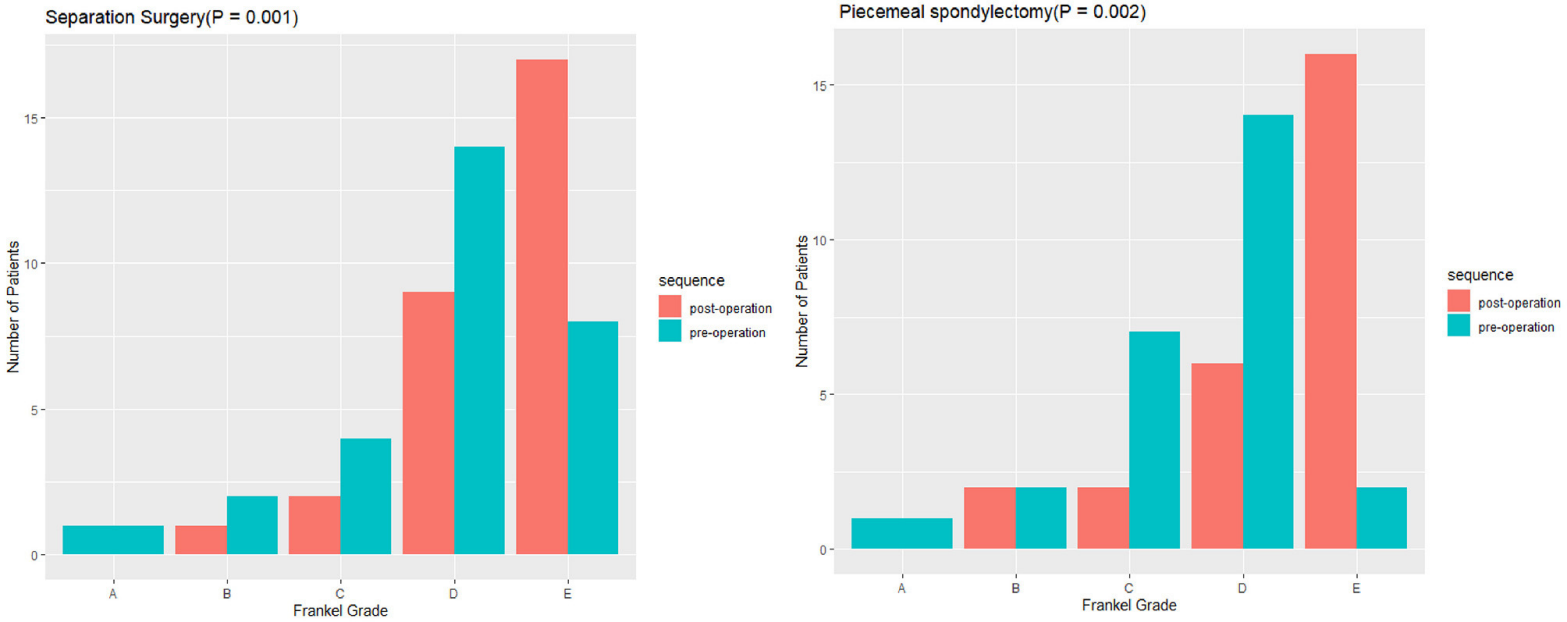
Comparisons of preoperative and postoperative neurological statuses between piecemeal spondylectomy and separation surgery.

Statistically significant differences in operative and perioperative factors were found between the two groups (Table 2). The piecemeal spondylectomy group had significantly higher blood loss (1784.62 ± 833.64 ml) than the separation surgery group (1235.52 ± 307.38 ml). The mean duration of operation was longer in the piecemeal spondylectomy group (4.76 ± 0.93 h) than in the separation group (3.73 ± 1.15) (Figure 1). No significant difference was found in preoperative and postoperative Frankel grades between the two groups. However, significant improvement in neurological status was detected in both groups (piecemeal spondylectomy group, *P* = 0.002; separation surgery, *P* = 0.001) after surgical procedures compared with before surgical procedures (Figure 2). Notably, one patient in the piecemeal spondylectomy group had neurological deterioration (from Frankel D to Frankel B) after surgery.

**Table 2:** Comparisons of perioperative and postoperative data between piecemeal spondylectomy and separation surgery.

	**Separation surgery**	**Piecemeal spondylectomy**	***P*-values**
Blood loss (ml)	1165.52 ± 307.38	1784.62 ± 833.64	0.005
Operative time (h)	3.73 ± 1.15	4.76 ± 0.93	0.001^*^
Postoperative Frankel grade			0.849
A	0 (0.00%)	0 (0.00%)	
B	1 (3.45%)	2 (7.69%)	
C	2 (6.90%)	2 (7.69%)	
D	9 (31.03%)	6 (23.08%)	
E	17 (58.62%)	16 (61.54%)	
Complications			0.029^*^
None	28 (96.5%)	22 (84.62%)	
Wound infection	1 (3.45%)	3 (11.54%)	
Pneumonia	0 (0.00%)	1 (3.85%)	
Cerebrospinal fluid leakage	0 (0.00%)	2 (7.69%)	
Overall survival			0.064
6 months	96.40%	69.20%	
1 year	80.90%	47.20%	
2 year	43.90%	39.30%	
Progression free survival			0.001^*^
6 months	96.40%	65.40%	
1 year	73.50%	33.90%	
2 year	35.70%	11.60%	

* *Statistically significant*.

**Figure 3:**
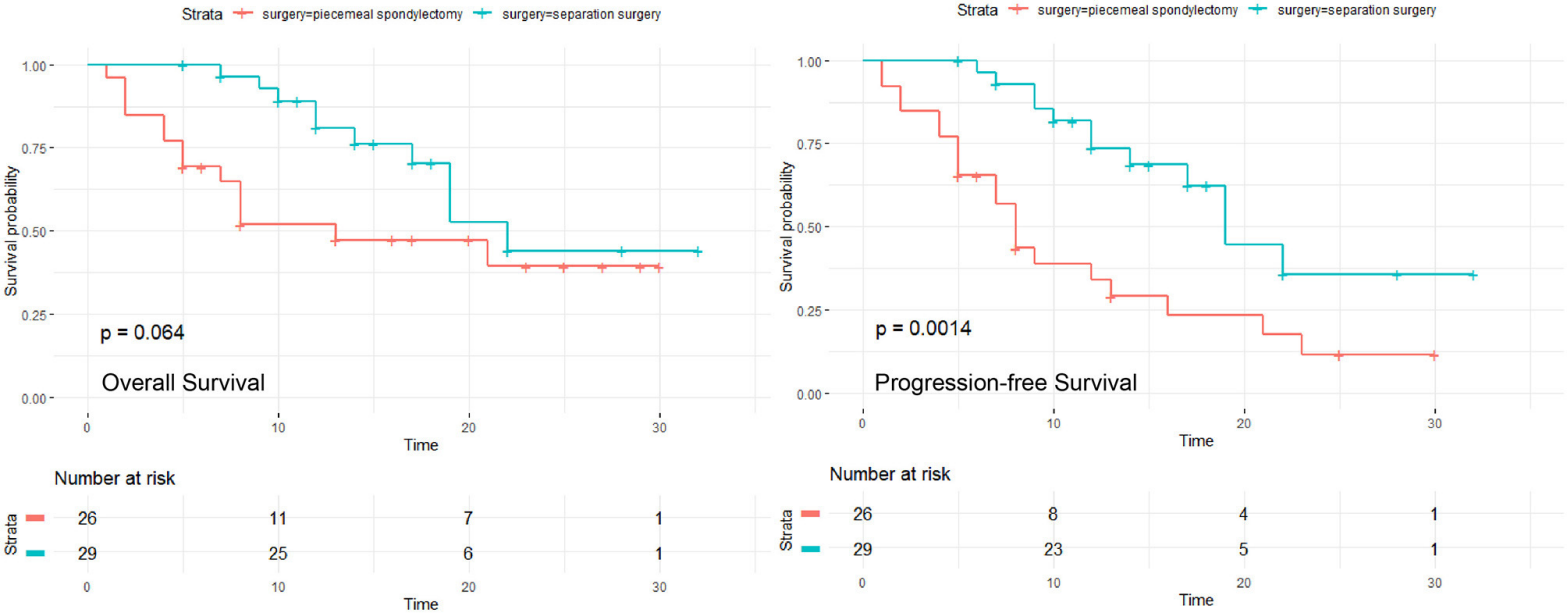
Comparisons of OS and PFS between piecemeal spondylectomy and separation surgery.

The median follow-up time was 13 months (range, 1–30 months), and 20 (76.92%) patients had local recurrence in the piecemeal spondylectomy group; at the last follow-up date, 6 (23.08%) patients died of the disease. The median follow-up time was 13 months (range, 5–32 months), and 12 (41.38%) patients developed local recurrence; 10 (34.48%) patients died at the end of follow-up in the separation surgery group. The 6-month, 1-year, and 2-year OS and PFS of both groups are listed in Table 2. Kaplan–Meier survival analysis was applied to estimate the OS and PFS in the two groups (Figure 3). The results revealed no significant difference in OS between the two groups (*P* = 0.064). However, the Kaplan–Meier curve of PFS indicated that the patients in the separation surgery group experienced less local recurrence than those in the piecemeal spondylectomy group (1-year rate of local recurrence: 13.79% in the separation surgery group vs. 23.08% in the piecemeal spondylectomy group; *P* = 0.0014).

**Figure 4:**
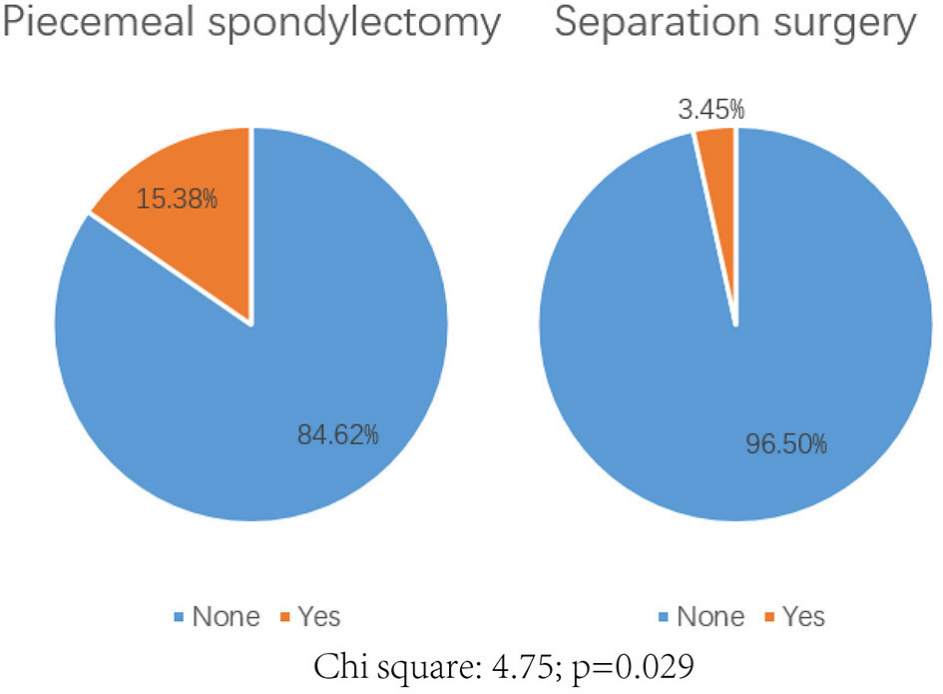
Comparison of the complication rate postoperatively between piecemeal spondylectomy and separation surgery. Chi-square: 4.75; *p* = 0.029.

A total of 7 (12.72%) patients experienced complications postoperatively (Table 2). No radiotherapy complication such as radiation myelopathy, vertebral compressive fractures (VCF), osteoradionecrosis or postradiation sarcomas was observed in all patients. Significant differences were detected in the development of complications between these two groups (*P* = 0.029, Figure 4). Only one patient in the separation group developed wound infection and received antibiotic therapy, but three patients in the piecemeal spondylectomy group experienced wound infection without postoperative revision. Two patients had cerebrospinal fluid leakage, which was treated conservatively, and one patient developed pneumonia during the inpatient period after surgery. Moreover, postoperative imaging data analyses showed no hardware failure in our study cohort, and no patient required revision surgery in the postoperative period.

## Discussion

In this study, we retrospectively reviewed 55 patients with spinal metastasis who underwent piecemeal spondylectomy or separation surgery in our institute. The surgical outcomes were evaluated, and the operative parameters were compared between both groups.

**Figure 5:**
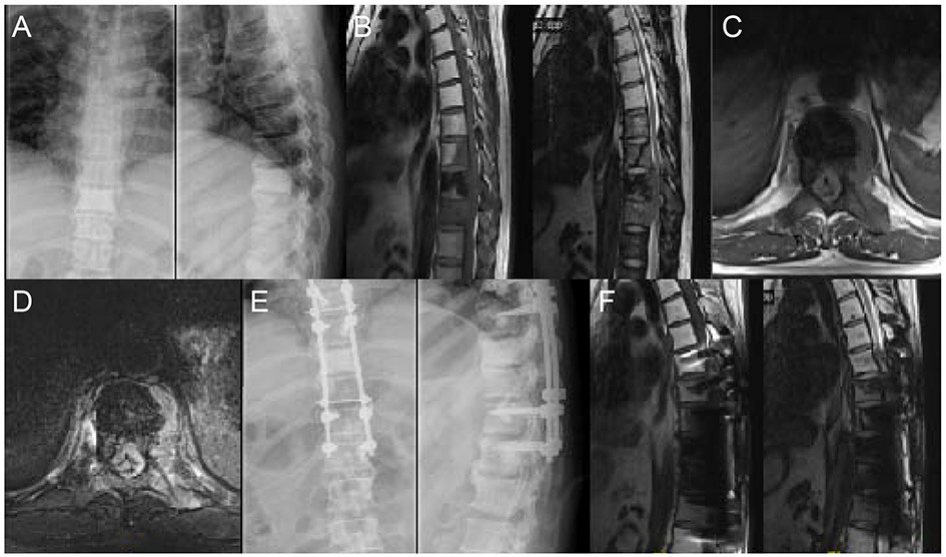
A 48 years old man who underwent surgery of lung cancer 6 years ago complained of increasing osphyalgia during the previous 1 month and was found to have metastatic lesion in T8-10; His preoperative Frankel grade was D. **(A)** Preoperative anteroposterior and lateral radiograph revealed abnormal bone mineral density in T9. **(B–D)** Sagittal and axial magnetic resonance imaging (MRI) showing signal intensity abnormalities in T8-10 and a pathological fracture of T9. **(E)** Postoperative anteroposterior and lateral radiograph. **(F)** Postoperative sagittal MRI.

Although there have been many reports on total spondylectomy and separation surgery for spinal tumors (6, 9, 11, 13, 18–22), most of them have focused on the single treatment modality. With the development of medical technology, the treatment strategies of metastatic spinal tumors have changed over the years. The emergence of stereotactic radiotherapy has led to the evolution of separation surgery (Figure 5). Molding et al. (12) first applied radiosurgery to 21 patients with spinal metastases after surgical decompression and instrumentation. They found that the 1-year local recurrence rate was 9.5%, and the overall local control rate was 81%; furthermore, patients who received a higher radiation dose showed a better local control rate. In addition, Laufer et al. (22) retrospectively analyzed 186 patients with metastatic epidural spinal cord compression who were treated with separation surgery and found that 16.3% of those patients had local recurrence 1 year after treatment. Moreover, Bate et al. (23) showed that the 1-year local recurrence rate was 9% in 69 patients who underwent separation surgery with (21 patients) or without (48 patients) SRS. In our study, the 1-year local recurrence rate was 13.79%, which is similar to that in previous reports. A careful preoperative assessment is required to provide a better choice of therapeutic options. The neurologic, oncologic, mechanical, and systemic (NOMS) decision framework involving multiple considerations has been proposed to optimize the combination of surgical procedure and radiosurgery (24). According to the NOMS framework, indications for separation surgery are radio-resistant tumors with high-grade epidural spinal cord compression. In addition, patients with pathological fractures or spinal instability may also be suitable for separation surgery (24).

**Figure 6:**
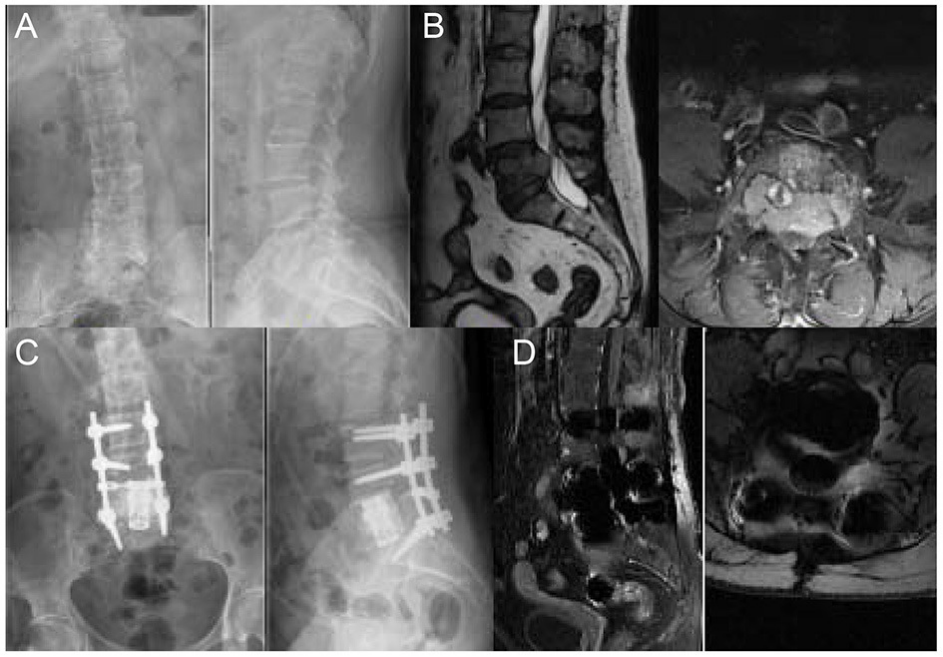
A 59 years old woman with breast cancer bone metastasis in L5; Her preoperative Frankel grade was D. **(A)** Preoperative anteroposterior and lateral radiograph revealed uneven density in L5. **(B)** Preoperative sagittal T2-weighted and axial T1-weighted MRI revealed the metastatic lesion with an intraspinal lesion. The protrusion of soft tissue resulted in compression of the dura and canal stenosis. **(C)** Postoperative anteroposterior and lateral radiograph showed that posterior spinal fusion with anterior reconstruction was performed. **(D)** Postoperative sagittal T1-weighted MRI.

According to the classification of GSTSG, the surgical technique of spondylectomy can be performed either in an en bloc or piecemeal manner (Figure 6). Previous studies have demonstrated the differences in surgical outcomes between piecemeal spondylectomy and other surgical procedures. For example, Kato et al. (25) compared outcomes between en bloc resection and piecemeal resection in the treatment of spinal metastases from thyroid cancer and found that the overall 5- and 10-year survival rates of patients who underwent en bloc resection were respectively 84 and 52%, which are significantly higher than those of patients who underwent piecemeal resection (5-year survival: 50%; 10-year survival: 8%); in the en bloc resection group, local recurrence was noted in only one patient, whereas almost all the patients who received piecemeal resection showed local recurrence and had reduced quality of life. Although total en bloc spondylectomy is considered an ideal choice for patients with solitary spinal metastasis, the complex surgical procedure has limited the practicality of this method. In addition, Ohashi et al. showed that 72.2% of patients experienced distant dissemination after en bloc resection in the long-term follow-up (>48 months) (26), indicating that en bloc resection is unable to prevent distant relapse despite the low rate of local recurrence. Moreover, compared with debulking, en bloc surgery showed similar survival outcomes, neurological improvements, and complication rates (27). Therefore, radical resection of spinal tumors in a piecemeal manner remains a more frequent surgical option (28).

In this study, we compared the surgical outcomes between piecemeal spondylectomy and separation surgery in the treatment of spinal metastasis. The separation surgery group (1165.52 ± 307.38 ml) had significantly less intraoperative blood loss than the piecemeal spondylectomy group (1784.62 ± 833.64 ml). Substantial blood loss could lead to increased requirement of allogeneic blood transfusion and even threaten life, and intraoperative hemorrhaging makes it difficult to obtain a clear surgical field, thereby leading to increased surgical risk, more perioperative complications, and delayed recovery. In addition, we found that patients in both groups showed improved neurological function after surgery, except for one patient in the piecemeal spondylectomy group who had neurological deterioration. However, the piecemeal spondylectomy group was associated with a higher complication rate (15.38 vs. 3.45%) and required longer operative time than the separation surgery group (4.76 ± 0.93 vs. 3.73±1.15 h). Radiation myelopathy and vertebral compressive fractures (VCF) were considered to be the mainly complications after SRS (29, 30). Other complications include poor wound healing, osteoradionecrosis and postradiation sarcomas. Due to the relatively low incidence and late presentation of SRS associated complication (30, 31), only three cases of poor surgical wound healing was observed in this study. Moreover, in survival analysis, we found no significant difference in OS between the two groups, but patients in the separation group had better PFS than those in the spondylectomy group. In the piecemeal method, the surgical tumor margins would inevitably become intralesional and influence the OS and local recurrence in spinal metastasis patients. In the resection of solitary metastasis of other organs, an R0 resection is independently associated with improved OS (32–35). However, the surgical management of spinal metastasis is largely not curable, meaning that the main purpose of surgery is to stabilize the unstable spine, regain neurological function, provide durable tumor control and improve quality of life. Previous studies also have demonstrated that spondylectomy was not associated with OS (21, 24, 26, 27). Despite we wanted to proof that the complete resection also present strong prognostic factor in spinal metastsis, no significant difference in OS was observed between two groups in this study. It is probably due to the uncontrolled systemic disease in patients. Notably, studies have demonstrated that contaminated surgical margins and intralesional resection increase the risk of local recurrence (27, 36, 37), and SRS can provide excellent local control after surgery for spinal metastasis (22, 23, 38–40). Since no literature has reported that radiotherapy is required after piecemeal spondylectomy, patients with negative surgical margins received did not undergone postoperative radiotherapy. Only six patients with positive margins in piecemeal resection group received conventional External Beam Radiation Therapy (cEBRT). It seems that one of the reason for poorer PFS in the piecemeal spondylectomy group is possibly caused by the lack of postoperative SRS as compared to separation surgery group. As a whole, these factors mentioned above might have led to the difference in PFS between the separation surgery cohort and the piecemeal spondylectomy cohort.

There are several limitations in our study. This retrospective study was performed at a single institution, and the sample size was small; therefore, the generalizability of our results is limited. In addition, this study had a limited follow-up duration because of the limited life expectancy in patients with spinal metastasis. Our results indicated that separation surgery combined with SRS could provide better local control and was associated with reduced surgical trauma compared with piecemeal spondylectomy. Regrettably, the separation surgery combined with SRS is a relatively recent technique in the field of metastatic spinal tumor and the period of piecemeal resection was conducted a few years earlier. As a result, few patients in piecemeal resection group received postoperative SRS. Due to the limitation of our current cohort, we were unable to test whether the postoperative SRS is the key factor that lead to the poorer PFS in piecemeal spondylectomy group. Further prospective studies with a larger sample size are required to validate the conclusion of our study to guide the choice of clinical treatment for patients with spinal metastasis.

## Conclusion

This retrospective study demonstrated that separation surgery and piecemeal spondylectomy resulted in equal improvements in neurological function in patients with spinal metastasis. However, separation surgery was associated with less blood loss, shorter intraoperative time, lower complication rate, and better local control. Therefore, separation surgery may be a good alternative to piecemeal spondylectomy for patients with spinal metastatic disease.

## Data Availability Statement

The raw data supporting the conclusions of this article will be made available by the authors, without undue reservation.

## Ethics Statement

The studies involving human participants were reviewed and approved by Medical Ethical Committee of Shanghai Cancer Center. The patients/participants provided their written informed consent to participate in this study. Written informed consent was obtained from the individual(s) for the publication of any potentially identifiable images or data included in this article.

## Author Contributions

WY, MC, and LX conceived and designed the study. WC, ZS, MF, and YJ collected the clinical data. SW, JZ, and TH performed the statistical analysis. LX draft the manuscript. WY and MC revised the paper. All authors contributed to the article and approved the submitted version.

## Conflict of Interest

The authors declare that the research was conducted in the absence of any commercial or financial relationships that could be construed as a potential conflict of interest.

## Publisher's Note

All claims expressed in this article are solely those of the authors and do not necessarily represent those of their affiliated organizations, or those of the publisher, the editors and the reviewers. Any product that may be evaluated in this article, or claim that may be made by its manufacturer, is not guaranteed or endorsed by the publisher.
